# 4-(4-Chloro­phen­yl)-7,7-dimethyl-7,8-dihydro-4*H*-1-benzopyran-2,5(3*H*,6*H*)-dione

**DOI:** 10.1107/S1600536809051320

**Published:** 2009-12-04

**Authors:** Hao Shi

**Affiliations:** a The College of Pharmaceutical Science, Zhejiang University of Technology, Hangzhou 310014, People’s Republic of China

## Abstract

The title compound, C_17_H_17_ClO_3_, has been synthesized by the reaction of *p*-chloro­benzaldehyde, isopropyl­idene malonate and 5,5-dimethyl­cyclo­hexane-1,3-dione with triethyl­benzyl­ammonium chloride in water as a green solvent. The six membered pyran­one ring of the hexa­hydro­coumarin system has a screw-boat conformation while the dimethyl­cyclo­hexenone system has a distorted envelope conformation. The dihedral angle between the least-squares planes of the coumarin ring system and the benzene ring is 85.64 (9)°.

## Related literature

For applications of coumarin derivatives, see: Wang *et al.* (1999 ▶); Yang (2001 ▶). For related structures, see: Itoh & Kanemasa (2003 ▶); Itoh *et al.* (2005 ▶). For ring puckering parameters, see: Cremer & Pople (1975 ▶).
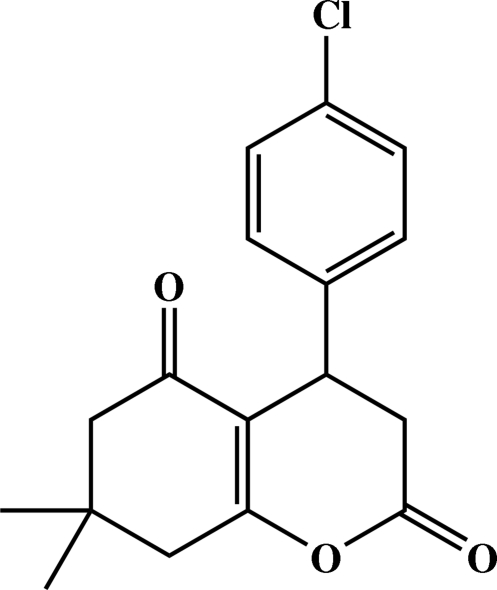


## Experimental

### 

#### Crystal data

C_17_H_17_ClO_3_
*M* *_r_* = 304.76
Monoclinic, 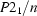

*a* = 11.9005 (12) Å
*b* = 5.7971 (8) Å
*c* = 22.608 (2) Å
β = 93.972 (1)°
*V* = 1555.9 (3) Å^3^
*Z* = 4
Mo *K*α radiation
μ = 0.25 mm^−1^
*T* = 298 K
0.48 × 0.39 × 0.34 mm

#### Data collection

Bruker SMART CCD area-detector diffractometer
Absorption correction: multi-scan (*SADABS*; Bruker, 1999 ▶) *T*_min_ = 0.889, *T*_max_ = 0.919
7519 measured reflections
2789 independent reflections
1585 reflections with *I* > 2σ(*I*)
*R*_int_ = 0.034

#### Refinement

*R*[*F* ^2^ > 2σ(*F* ^2^)] = 0.062
*wR*(*F* ^2^) = 0.186
*S* = 1.05
2789 reflections
192 parameters
H-atom parameters constrained
Δρ_max_ = 0.49 e Å^−3^
Δρ_min_ = −0.51 e Å^−3^

### 

Data collection: *SMART* (Bruker, 1999 ▶); cell refinement: *SAINT* (Bruker, 1999 ▶); data reduction: *SAINT*; program(s) used to solve structure: *SHELXS97* (Sheldrick, 2008 ▶); program(s) used to refine structure: *SHELXL97* (Sheldrick, 2008 ▶); molecular graphics: *ORTEP-3* (Farrugia, 1997 ▶); software used to prepare material for publication: *SHELXL97*.

## Supplementary Material

Crystal structure: contains datablocks I, global. DOI: 10.1107/S1600536809051320/bh2260sup1.cif

Structure factors: contains datablocks I. DOI: 10.1107/S1600536809051320/bh2260Isup2.hkl

Additional supplementary materials:  crystallographic information; 3D view; checkCIF report

## Figures and Tables

**Fig. 1.**
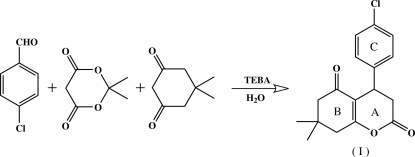
The preparation of the title compound.

**Fig. 2.**
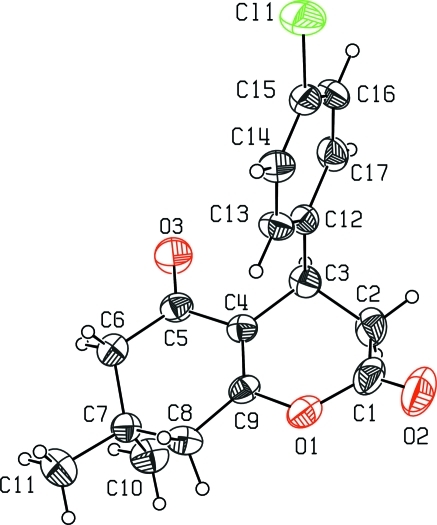
Structure of the title compound, showing 30% probability displacement ellipsoids with atomic numbering scheme.

## supplementary crystallographic information

### Comment

Coumarin is an important chemical having unique characteristics. It is widely used in hand soaps, detergents, lotions and laser dyes (Wang *et al.*, 1999). Coumarin and some of its derivatives have been tested in pharmacology for treatment of HIV (Yang, 2001). To obtain coumarin in a more environment friendly way, water was used as a green solvent in the synthesis of the title compound (Fig.1 and *Experimental*).

In the molecule of the title compound (Fig. 2), the two six membered rings of the hexahydrocoumarin system are not planar, having screw-boat and envelope conformations respectively: the pyranone ring *A* (O1/C1···C4/C9) adopts the screw-boat conformation with puckering parameters (Cremer & Pople, 1975) *Q*= 0.430 (5) Å, θ= 61.8 (5)° and φ = 134.7 (6)°; the ring *B* (C4···C9) exists in a distorted envelope conformation [*Q* = 0.408 (4) Å, θ= 127.0 (6)° and φ= 343.4 (7)°)] with C7 displaced by 0.558 (5) Å from the plane of the other ring atoms. Ring *C* (C12···C17) is a benzene ring, which makes a dihedral angle of 85.64 (9)° with the least-squares plane of the coumarin ring. The analogue of the title compound including Br in place of Cl has been reported and an enantiomerically pure crystal characterized by X-ray diffraction (Itoh & Kanemasa, 2003; Itoh *et al.*, 2005).

### Experimental

A mixture of 4-chlorobenzaldehyde (100 mmol), 5,5-dimethyl-1,3-cyclohexanedione (100 mmol), isopropylidene malonate (100 mmol), triethylbenzylammonium chloride (TEBA) (15 mmol) and 400 mL of water was transferred into a flask connected with refluxing equipment (Fig. 1). After stirring at 345 K (72°C) for 5 h, the reaction mixture was cooled to room temperature, the precipitated product was filtered and recrystallized with ethanol to give the title compound. Crystals suitable for X-ray structure analysis were obtained by slow evaporation from a solution of isopropyl alcohol at room temperature.

### Refinement

All H atoms were placed in geometrical positions and constrained to ride on their parent atoms with C—H distances in the range 0.93–0.98 Å. They were treated as riding atoms, with *U*_iso_(H) = 1.5*U*_eq_(carrier C) for methyl groups and *U*_iso_(H) = 1.2*U*_eq_(carrier C) for other H atoms.

### Crystal data

**Table:**

C_17_H_17_ClO_3_	*F*(000) = 640
*M**_r_* = 304.76	*D*_x_ = 1.301 Mg m^−^^3^
Monoclinic, *P*2_1_/*n*	Mo *K*α radiation, λ = 0.71073 Å
Hall symbol: -P 2yn	Cell parameters from 1909 reflections
*a* = 11.9005 (12) Å	θ = 2.7–23.0°
*b* = 5.7971 (8) Å	µ = 0.25 mm^−^^1^
*c* = 22.608 (2) Å	*T* = 298 K
β = 93.972 (1)°	Prism, colorless
*V* = 1555.9 (3) Å^3^	0.48 × 0.39 × 0.34 mm
*Z* = 4	

### Data collection

**Table:**

Bruker SMART CCD area-detector diffractometer	2789 independent reflections
Radiation source: fine-focus sealed tube	1585 reflections with *I* > 2σ(*I*)
graphite	*R*_int_ = 0.034
φ and ω scans	θ_max_ = 25.2°, θ_min_ = 1.8°
Absorption correction: multi-scan (*SADABS*; Bruker, 1999)	*h* = −12→14
*T*_min_ = 0.889, *T*_max_ = 0.919	*k* = −6→6
7519 measured reflections	*l* = −27→20

### Refinement

**Table:**

Refinement on *F*^2^	Primary atom site location: structure-invariant direct methods
Least-squares matrix: full	Secondary atom site location: difference Fourier map
*R*[*F*^2^ > 2σ(*F*^2^)] = 0.062	Hydrogen site location: inferred from neighbouring sites
*wR*(*F*^2^) = 0.186	H-atom parameters constrained
*S* = 1.05	*w* = 1/[σ^2^(*F*_o_^2^) + (0.0667*P*)^2^ + 1.4872*P*] where *P* = (*F*_o_^2^ + 2*F*_c_^2^)/3
2789 reflections	(Δ/σ)_max_ < 0.001
192 parameters	Δρ_max_ = 0.49 e Å^−^^3^
0 restraints	Δρ_min_ = −0.51 e Å^−^^3^
0 constraints	

### Fractional atomic coordinates and isotropic or equivalent isotropic displacement parameters (Å^2^)

**Table:**

	*x*	*y*	*z*	*U*_iso_*/*U*_eq_	
Cl1	−0.43477 (9)	0.5886 (3)	0.12137 (6)	0.1069 (6)	
O1	0.1778 (2)	0.6708 (5)	0.18437 (12)	0.0681 (8)	
O2	0.1132 (3)	0.5408 (7)	0.26641 (15)	0.1127 (13)	
O3	0.0220 (3)	1.3042 (5)	0.07706 (14)	0.0936 (10)	
C1	0.1180 (4)	0.7039 (10)	0.2336 (2)	0.0765 (13)	
C2	0.0708 (4)	0.9367 (9)	0.24091 (17)	0.0810 (14)	
H2A	0.1305	1.0395	0.2559	0.097*	
H2B	0.0149	0.9303	0.2701	0.097*	
C3	0.0160 (3)	1.0357 (7)	0.18295 (16)	0.0598 (10)	
H3	0.0049	1.2017	0.1883	0.072*	
C4	0.0969 (3)	1.0017 (6)	0.13547 (15)	0.0505 (9)	
C5	0.0888 (3)	1.1459 (7)	0.08223 (17)	0.0602 (10)	
C6	0.1584 (3)	1.0799 (8)	0.03203 (18)	0.0751 (12)	
H6A	0.1720	1.2168	0.0089	0.090*	
H6B	0.1156	0.9727	0.0064	0.090*	
C7	0.2710 (3)	0.9703 (7)	0.05132 (16)	0.0580 (10)	
C8	0.2544 (3)	0.7783 (7)	0.09489 (18)	0.0631 (10)	
H8A	0.2291	0.6416	0.0731	0.076*	
H8B	0.3265	0.7424	0.1155	0.076*	
C9	0.1725 (3)	0.8325 (6)	0.13923 (16)	0.0518 (9)	
C10	0.3488 (4)	1.1568 (8)	0.0813 (2)	0.0812 (13)	
H10A	0.3137	1.2211	0.1145	0.122*	
H10B	0.3616	1.2767	0.0532	0.122*	
H10C	0.4195	1.0883	0.0947	0.122*	
C11	0.3302 (4)	0.8824 (9)	−0.0017 (2)	0.0955 (15)	
H11A	0.4020	0.8187	0.0115	0.143*	
H11B	0.3409	1.0077	−0.0285	0.143*	
H11C	0.2849	0.7653	−0.0217	0.143*	
C12	−0.0981 (3)	0.9254 (6)	0.16736 (15)	0.0524 (9)	
C13	−0.1075 (3)	0.7218 (7)	0.13616 (17)	0.0585 (10)	
H13	−0.0428	0.6531	0.1235	0.070*	
C14	−0.2100 (3)	0.6170 (7)	0.12320 (17)	0.0636 (10)	
H14	−0.2141	0.4778	0.1027	0.076*	
C15	−0.3048 (3)	0.7190 (8)	0.14062 (17)	0.0638 (11)	
C16	−0.3001 (3)	0.9189 (9)	0.17169 (18)	0.0719 (12)	
H16	−0.3658	0.9854	0.1838	0.086*	
C17	−0.1966 (3)	1.0245 (7)	0.18543 (17)	0.0666 (11)	
H17	−0.1932	1.1618	0.2068	0.080*	

### Atomic displacement parameters (Å^2^)

**Table:**

	*U*^11^	*U*^22^	*U*^33^	*U*^12^	*U*^13^	*U*^23^
Cl1	0.0534 (6)	0.1496 (13)	0.1172 (11)	−0.0213 (7)	0.0011 (6)	0.0253 (9)
O1	0.0610 (16)	0.0693 (18)	0.0721 (18)	−0.0014 (13)	−0.0099 (14)	0.0155 (15)
O2	0.128 (3)	0.132 (3)	0.075 (2)	−0.020 (2)	−0.0128 (19)	0.040 (2)
O3	0.110 (2)	0.074 (2)	0.097 (2)	0.0438 (19)	0.0137 (19)	0.0108 (18)
C1	0.072 (3)	0.101 (4)	0.054 (3)	−0.020 (3)	−0.017 (2)	0.008 (3)
C2	0.081 (3)	0.110 (4)	0.050 (2)	−0.024 (3)	−0.004 (2)	−0.018 (3)
C3	0.064 (2)	0.057 (2)	0.059 (2)	−0.0037 (19)	0.0033 (18)	−0.0186 (19)
C4	0.0476 (18)	0.048 (2)	0.055 (2)	−0.0025 (16)	−0.0024 (15)	−0.0082 (18)
C5	0.060 (2)	0.053 (2)	0.067 (3)	0.0078 (19)	−0.0015 (19)	−0.001 (2)
C6	0.073 (3)	0.091 (3)	0.061 (2)	0.014 (2)	0.002 (2)	0.007 (2)
C7	0.055 (2)	0.062 (2)	0.057 (2)	0.0042 (18)	0.0022 (17)	−0.002 (2)
C8	0.051 (2)	0.055 (2)	0.084 (3)	0.0043 (18)	0.0070 (19)	0.001 (2)
C9	0.0462 (19)	0.051 (2)	0.057 (2)	−0.0052 (17)	−0.0076 (16)	0.0054 (19)
C10	0.073 (3)	0.069 (3)	0.101 (3)	−0.013 (2)	0.002 (2)	0.005 (3)
C11	0.092 (3)	0.110 (4)	0.087 (3)	0.014 (3)	0.025 (3)	−0.007 (3)
C12	0.056 (2)	0.054 (2)	0.047 (2)	0.0089 (17)	0.0058 (16)	−0.0044 (18)
C13	0.0450 (19)	0.062 (2)	0.068 (2)	0.0058 (17)	0.0046 (17)	−0.014 (2)
C14	0.053 (2)	0.066 (3)	0.071 (3)	−0.0035 (19)	−0.0004 (18)	−0.002 (2)
C15	0.050 (2)	0.081 (3)	0.061 (2)	−0.002 (2)	0.0028 (18)	0.018 (2)
C16	0.053 (2)	0.094 (3)	0.071 (3)	0.020 (2)	0.022 (2)	0.018 (3)
C17	0.077 (3)	0.064 (3)	0.060 (2)	0.015 (2)	0.015 (2)	−0.004 (2)

### Geometric parameters (Å, °)

**Table:**

Cl1—C15	1.749 (4)	C7—C10	1.549 (5)
O1—C1	1.374 (5)	C8—C9	1.480 (5)
O1—C9	1.384 (4)	C8—H8A	0.9700
O2—C1	1.206 (5)	C8—H8B	0.9700
O3—C5	1.214 (4)	C10—H10A	0.9600
C1—C2	1.475 (7)	C10—H10B	0.9600
C2—C3	1.534 (6)	C10—H10C	0.9600
C2—H2A	0.9700	C11—H11A	0.9600
C2—H2B	0.9700	C11—H11B	0.9600
C3—C4	1.503 (5)	C11—H11C	0.9600
C3—C12	1.520 (5)	C12—C13	1.376 (5)
C3—H3	0.9800	C12—C17	1.392 (5)
C4—C9	1.330 (5)	C13—C14	1.375 (5)
C4—C5	1.463 (5)	C13—H13	0.9300
C5—C6	1.501 (5)	C14—C15	1.357 (5)
C6—C7	1.519 (5)	C14—H14	0.9300
C6—H6A	0.9700	C15—C16	1.354 (6)
C6—H6B	0.9700	C16—C17	1.391 (6)
C7—C8	1.509 (5)	C16—H16	0.9300
C7—C11	1.520 (6)	C17—H17	0.9300
			
C1—O1—C9	120.2 (3)	C9—C8—H8B	108.7
O2—C1—O1	116.0 (5)	C7—C8—H8B	108.7
O2—C1—C2	127.8 (5)	H8A—C8—H8B	107.6
O1—C1—C2	116.2 (4)	C4—C9—O1	122.9 (3)
C1—C2—C3	112.9 (3)	C4—C9—C8	126.0 (3)
C1—C2—H2A	109.0	O1—C9—C8	110.9 (3)
C3—C2—H2A	109.0	C7—C10—H10A	109.5
C1—C2—H2B	109.0	C7—C10—H10B	109.5
C3—C2—H2B	109.0	H10A—C10—H10B	109.5
H2A—C2—H2B	107.8	C7—C10—H10C	109.5
C4—C3—C12	112.6 (3)	H10A—C10—H10C	109.5
C4—C3—C2	107.8 (3)	H10B—C10—H10C	109.5
C12—C3—C2	111.1 (3)	C7—C11—H11A	109.5
C4—C3—H3	108.4	C7—C11—H11B	109.5
C12—C3—H3	108.4	H11A—C11—H11B	109.5
C2—C3—H3	108.4	C7—C11—H11C	109.5
C9—C4—C5	118.7 (3)	H11A—C11—H11C	109.5
C9—C4—C3	121.0 (3)	H11B—C11—H11C	109.5
C5—C4—C3	120.2 (3)	C13—C12—C17	117.7 (3)
O3—C5—C4	121.1 (4)	C13—C12—C3	121.3 (3)
O3—C5—C6	120.8 (4)	C17—C12—C3	121.0 (3)
C4—C5—C6	117.9 (3)	C14—C13—C12	121.9 (3)
C5—C6—C7	114.3 (3)	C14—C13—H13	119.1
C5—C6—H6A	108.7	C12—C13—H13	119.1
C7—C6—H6A	108.7	C15—C14—C13	119.2 (4)
C5—C6—H6B	108.7	C15—C14—H14	120.4
C7—C6—H6B	108.7	C13—C14—H14	120.4
H6A—C6—H6B	107.6	C16—C15—C14	121.2 (4)
C8—C7—C6	110.1 (3)	C16—C15—Cl1	120.2 (3)
C8—C7—C11	110.9 (3)	C14—C15—Cl1	118.6 (4)
C6—C7—C11	111.2 (3)	C15—C16—C17	119.8 (4)
C8—C7—C10	109.3 (3)	C15—C16—H16	120.1
C6—C7—C10	108.8 (3)	C17—C16—H16	120.1
C11—C7—C10	106.5 (3)	C16—C17—C12	120.2 (4)
C9—C8—C7	114.1 (3)	C16—C17—H17	119.9
C9—C8—H8A	108.7	C12—C17—H17	119.9
C7—C8—H8A	108.7		
			
C9—O1—C1—O2	169.1 (3)	C5—C4—C9—O1	−172.3 (3)
C9—O1—C1—C2	−12.9 (5)	C3—C4—C9—O1	3.8 (5)
O2—C1—C2—C3	−138.5 (4)	C5—C4—C9—C8	2.4 (5)
O1—C1—C2—C3	43.7 (5)	C3—C4—C9—C8	178.5 (3)
C1—C2—C3—C4	−48.0 (5)	C1—O1—C9—C4	−12.2 (5)
C1—C2—C3—C12	75.9 (4)	C1—O1—C9—C8	172.4 (3)
C12—C3—C4—C9	−97.0 (4)	C7—C8—C9—C4	16.7 (5)
C2—C3—C4—C9	26.0 (5)	C7—C8—C9—O1	−168.1 (3)
C12—C3—C4—C5	79.1 (4)	C4—C3—C12—C13	35.1 (5)
C2—C3—C4—C5	−157.9 (3)	C2—C3—C12—C13	−86.0 (4)
C9—C4—C5—O3	−179.1 (4)	C4—C3—C12—C17	−146.4 (3)
C3—C4—C5—O3	4.7 (5)	C2—C3—C12—C17	92.5 (4)
C9—C4—C5—C6	6.0 (5)	C17—C12—C13—C14	−0.3 (5)
C3—C4—C5—C6	−170.1 (3)	C3—C12—C13—C14	178.2 (4)
O3—C5—C6—C7	152.0 (4)	C12—C13—C14—C15	1.3 (6)
C4—C5—C6—C7	−33.1 (5)	C13—C14—C15—C16	−1.6 (6)
C5—C6—C7—C8	49.9 (5)	C13—C14—C15—Cl1	177.7 (3)
C5—C6—C7—C11	173.2 (4)	C14—C15—C16—C17	0.9 (6)
C5—C6—C7—C10	−69.9 (5)	Cl1—C15—C16—C17	−178.3 (3)
C6—C7—C8—C9	−41.4 (4)	C15—C16—C17—C12	0.0 (6)
C11—C7—C8—C9	−164.8 (3)	C13—C12—C17—C16	−0.3 (5)
C10—C7—C8—C9	78.1 (4)	C3—C12—C17—C16	−178.8 (3)
